# Flow Cytometric Characterization of T Cell Subsets and Microglia After Repetitive Mild Traumatic Brain Injury in Rats

**DOI:** 10.1007/s11064-017-2310-0

**Published:** 2017-06-15

**Authors:** Ruojing Bai, Huabin Gao, Zhaoli Han, Shan Huang, Xintong Ge, Fanglian Chen, Ping Lei

**Affiliations:** 1 Department of Geriatrics, Tianjin Medical University General Hospital, Tianjin Geriatrics Institute, Anshan Road No. 154, Tianjin, 300052 China; 2 Laboratory of Neuro-Trauma and Neurodegenerative Disorder, Tianjin Geriatrics Institute, Tianjin, 300052 China; 3 Key Laboratory of Post-Trauma Neuro-Repair and Regeneration in Central Nervous System, Ministry of Education, Tianjin, 300052 China; 4 Key Laboratory of Injury, Variation and Regeneration of Nervous System, Tianjin, 300052 China; 5 Laboratory of Neuro-Trauma, Tianjin Neurological Institute, Tianjin, 300052 China; 6 Department of Neurosurgery, Tianjin Neurological Institute General Hospital, Tianjin Medical University, Tianjin, 300052 China

**Keywords:** Repetitive mild traumatic brain injury, T cell subsets, Microglia

## Abstract

Although, there is growing awareness in the progressive neurodegeneration of chronic traumatic encephalopathy, changes of immune reactions remain equivocal at best. Thus, in a clinically relevant rat repetitive mild traumatic brain injury (rmTBI) model, some immunologic cells (T cell subsets, microglia) in the injured brain and peripheral blood were analyzed by flow cytometry and immunofluorescence. In the injured brain, CD3^+^ T cells showed a bimodal increase during 42 days post-injury (dpi). CD3^+^CD4^+^ T cells firstly increased and then decreased, while CD3^+^CD8^+^ T cells had reversed tendency. CD86^+^/CD11b^+^ M1-like microglia increased at 42 dpi and CD206^+^/CD11b^+^ M2-like microglia peaked at 7 dpi. In addition, peripheral immune suppression was implicated in the chronic phase after rmTBI. Taken together, the study provided useful information on long-term dynamic changes of some immune cells after rmTBI in rats.

## Introduction

Traumatic brain injury (TBI) is a major public health issue, which caused by road traffic collisions, assaults, and falls, etc. Before retired athletes and veterans of wars, chronic traumatic encephalopathy (CTE), a progressive neurodegeneration of the brain tissue or a result of repetitive mild traumatic brain injury (rmTBI) [1], was not considered to be a common phenomenon.

TBI elicits robust immune events that leads to axonal damage and secondary injury. However, changes of T cell subsets and microglia post-rmTBI remain incomplete. Thus, our study would preliminary descriptive analysis.

## Method and Material

All research were approved by institutional (Animal Care and Use Committee of Tianjin Medical University). All animals were maintained according to the EC Directive 86/609/EEC for animal experiments. And all investigators were blinded to the treatment groups during animal surgery, data collection and analysis.

### Animals

12 weeks old male Sprague–Dawley rats (n = 162, each weighing about 200 g), which were purchased from the Chinese Academy of Military Science (Beijing, China) and bred at Experimental Animal Laboratories of Tianjin Neurological Institute, with a 12 h light–dark cycle (lights on at 7:00 a.m. and off at 7:00 p.m.), a constant temperature (22 ± 2 °C) and relative humidity (55–60%). Rats were randomly divided into 7 groups: the sham group and 1/3/7/14/28/42 dpi groups, with 6 rats in each group.

### Controlled Cortical Impact (CCI) Induced Repetitive Mild Traumatic Brain Injury (rmTBI) Model

Rats were anesthetized with 10% chloride hydrate (3.5 ml/kg intraperitoneal injection) and were put under a stereotaxic frame. The circular craniotomy (diameter, 4 mm) was performed on the right parietal skull, with center of the drill located at 4.0 mm posterior from bregma and 3.0 mm lateral to the sagittal suture. The impact was induced using a pneumatic impact device (American Instruments, Richmond, VA USA) with the standard parameters for CCI rmTBI, including velocity at 3.6 m/s, tracel range 1.2 mm, dwell settings 2 s, and 24 h apart. And the removed skull was returned to its original position and the incision was suture-closed. Sham operations underwent the same anesthesia and were not exposed to injury.

### Flow Cytometry

Rats were deeply anesthetized with 10% chloral hydrate at corresponding 1/3/7/14/28/42 dpi groups.

After collecting peripheral blood samples in K2-EDTA vials (BD Biosciences, USA), it was diluted with phosphate buffered saline (PBS; Sigma–Aldrich, USA) and stratified with lympholyte-mammal (Sigma–Aldrich, USA). After density gradient centrifugation at 300*g* for 20 min at room temperature, peripheral blood mononuclear cells (PBMCs) were isolated from collected peripheral blood. Cells were purified by washing with PBS twice times.

Rats were transcardially perfused with ice-cold PBS. Then brains were removed as soon as possible and homogenized with syringe through a 70 µM cell strainer (BD Biosciences, USA) to acquire a single cell suspension. After centrifugation at 1500 rpm for 10 min at room temperature, cells were re-suspended in 30% percoll and 70% percoll (GE Healthcare, Little Chalfont, UK, diluted in HBSS: Hanks’ balanced salt solution; Life Technologies, Carlsbad, CA). After centrifugation at 400*g* for 20 min at room temperature, CNS mononuclear cells were at the mid-layer, which was between the 30 and 70% percoll interface. Cells were purified by washing with PBS twice times.

Mononuclear cells were stained with Rat T lymphocyte Cocktail, anti-rat CD3-APC, anti-rat CD4-PE, anti-rat CD8-FITC, and anti-rat CD11b-FITC, anti-rat CD45-PerCP, anti-rat CD86-PE, anti-rat CD206-APC (BD Biosciences, USA) following standard protocols and manufacturer’s instructions. Data were obtained using a FACSCalibur (BD Biosciences, USA) and analyzed with Flow Jo VX software.

### Immunofluorescence Procedures

For the immunofluorescence staining, rats were sacrificed by transcardiac perfusion with cold PBS followed by 4% paraformaldehyde at corresponding 1/7/14/28/42 dpi groups. The dissected injured brain were fixed in 4% paraformaldehyde for 24 h at 4 °C, and incubated in 30% sucrose for 48 h. After fixation, they were embedded in the optimum cutting temperature (OCT) medium (Sakura, Torrance, CA, USA) on dry ice, and stored at −80 °C immediately. A series of 40 µm coronal sections using a Microm HM550 cryostat were made on a cryostat at −20 °C and processed for immunofluorescence.

After air drying, all sections were treated with 3% bovine serum albumin for 30 min at 37 °C to block nonspecific staining, and incubated over night at 4 °C with the primary antibody: anti-Iba-1 (ab107159, 1:200; Abcam). After being rinsed by PBS (3 × 10 min), the slides were incubated for 2 h at room temperature with a 1:2000 dilution of anti-goat IgG secondary antibody (Invitrogen, Carlsbad, CA, USA). The nuclei were counterstained with DAPI (Sigma–Aldrich, USA) at room temperature. Sections were digitized under a 20× objective using a 3-CCD color video camera (Sony DXC-970MD, Japan) with an immunofluorescence microscope (Olympus IX81, Japan).Four separate slides (40 μm apart from each other) from each brain with each slide containing three randomly selected 200× fields from the lesion site were digitized. Image processing analysis and measurements were performed using Image J software (National Institute of Health, USA).

### Statistics Analysis

The data were expressed as mean ± SD. All statistical analyses were conducted using SPSS 22.0 and Graphpad Prism 5 software. p vales were calculated with the single-factorial analysis of variance (ANOVA) and Student’s t-test. A p-value of less than 0.05 was significant.

## Results

### Dynamic Changes of T Lymphocyte Subsets in the Brain After rmTBI in Rats

T lymphocyte subsets were characterized by the expression of cell surface markers: all T cells (CD3^+^), CD4^+^ or CD8^+^ T cells (CD3^+^CD4^+^ or CD3^+^CD8^+^) [2].

**Fig. 1:**
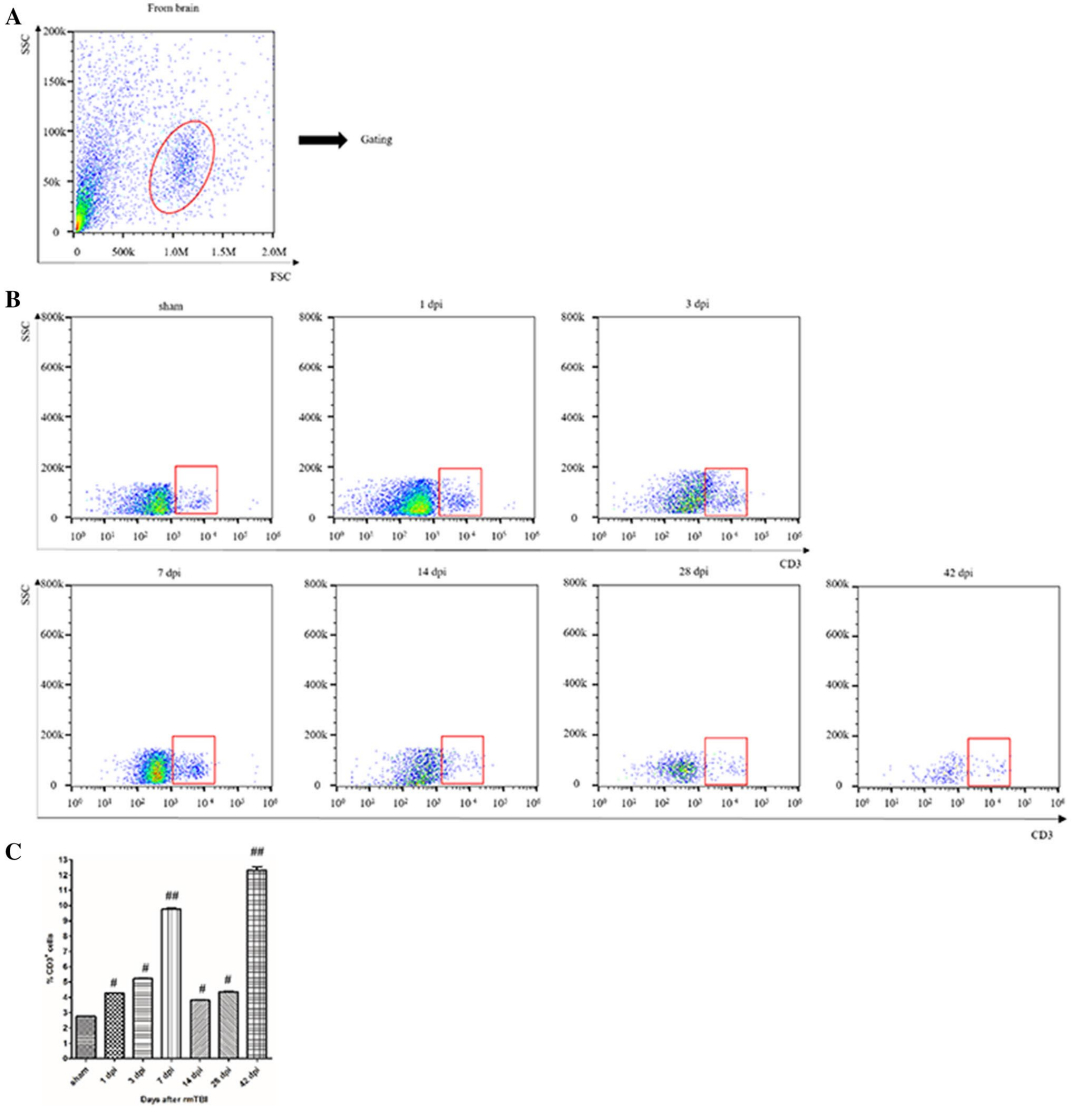
Analysis of T lymphocyte in the injured brain after rmTBI. **a** *Dot* plots of isolated immune cells in the brain, gated for live cell analysis. **b** Representative flow cytometry data for T cells (CD3^+^ cells) in the brain at the indicated days after rmTBI. **c** Graph illustrating quantitative data for accumulated T cells in the brain after rmTBI. n = 6 for each experiment. T cells: ^##^p < 0.01 at 7 and 42 dpi compared with sham; ^#^p < 0.05 at the others compared with sham

The gating strategy of live cell analysis was shown (Fig. 1a).The proportion of T cells significantly increased at 7 and 42 days post-injury (dpi) (Fig. 1b). Quantitative data for the percentage of T cells in CNS are shown in Fig. 1c.

**Fig. 2:**
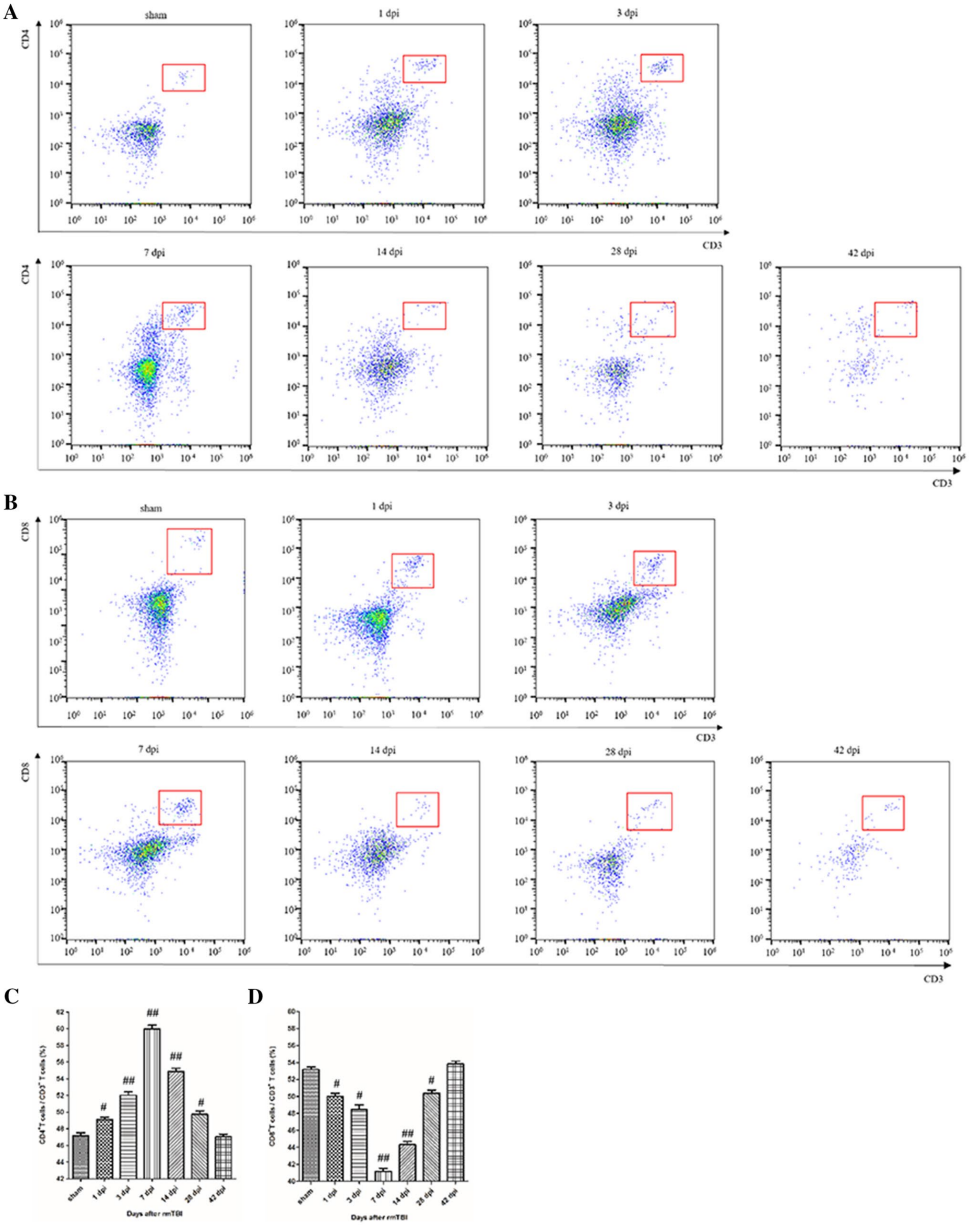
Analysis of T cell subsets in the injured brain after rmTBI. **a** Representative flow cytometry data for CD4^+^ T cells (CD3^+^ CD4^+^ cells) in the injured brain at the indicated days after rmTBI. **b** Representative flow cytometry data for CD8^+^ T cells (CD3^+^ CD8^+^ cells) in the injured brain at the indicated days after rmTBI. **c** Graph illustrating quantitative data for the percentages of CD4^+^ T cells in the brain at the indicated days after rmTBI. **d** Graph illustrating quantitative data for the percentages of CD8^+^ T cells in the brain at the indicated days after rmTBI. n = 6 for each experiment. CD4^+^ T cells: ^##^p < 0.01 at 3, 7, and 14 dpi compared with sham; ^#^p < 0.05 at 1 and 28 dpi compared with sham. CD8^+^ T cells: ^##^p < 0.01 at 7 and 14 dpi compared with sham; ^#^p < 0.05 at 1, 3, and 28 dpi compared with sham

The percentage of CD4^+^ T cells rose first during 7 dpi and gradually returned to the baseline (Fig. 2a). The percentage of CD8^+^ T cells decreased during 7 dpi, and returned to the baseline level at 42 dpi (Fig. 2b). Quantitative data for the percentage of CD4 + and CD8 + T cells in CNS are shown in Fig. 2c, d.

### Dynamic Changes of Microglia in the Brain After rmTBI in Rats

**Fig. 3:**
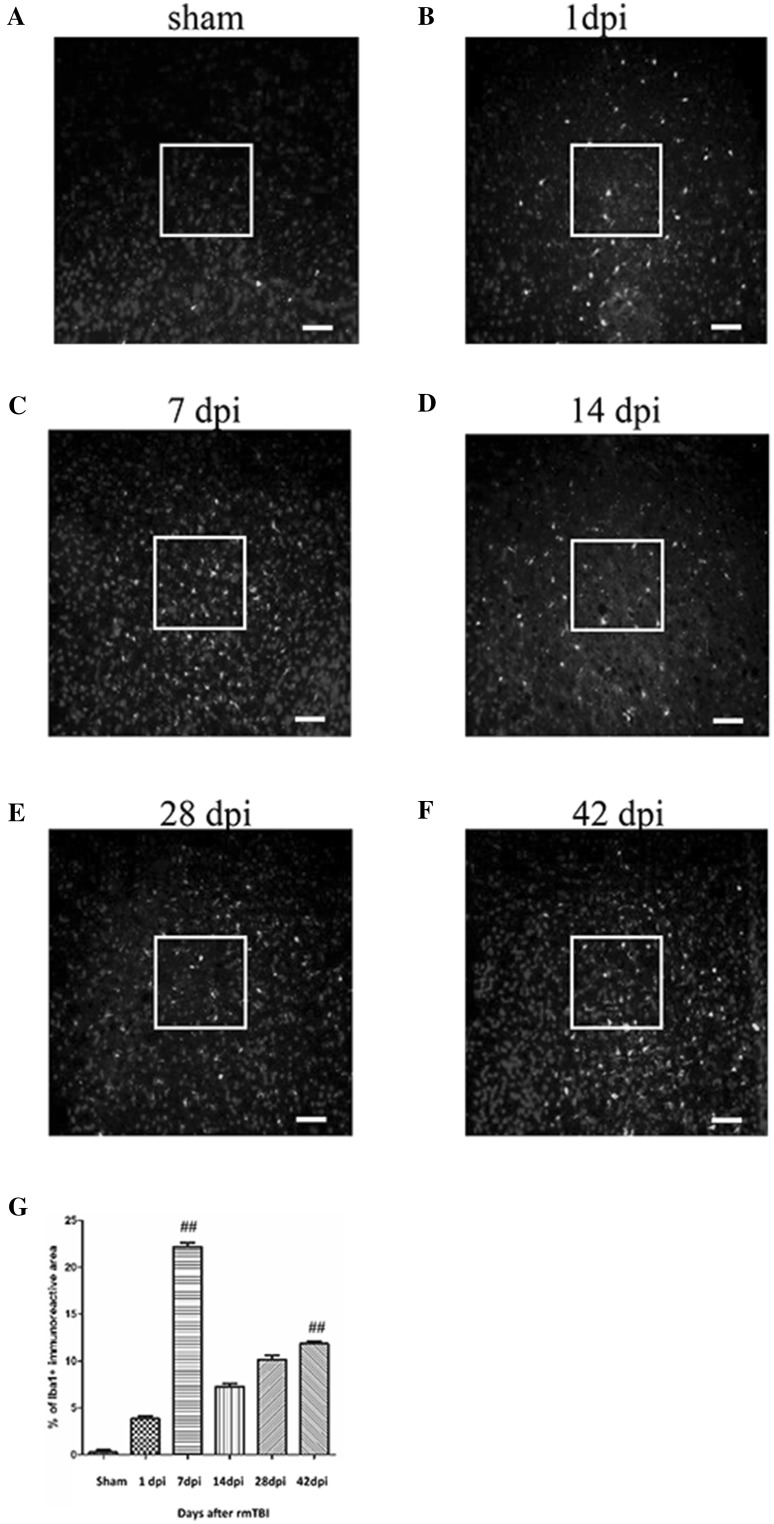
Immunofluorescence analysis of the Iba-1^+^ cells in the injured brain after rmTBI. Sections are stained of Iba-1 in sham (**a**) and at 1 dpi (**b**), 7 dpi (**c**), 14 dpi (**d**), 28 dpi (**e**) and 42 dpi (**f**). **g** The graph shows the percentages of Iba-1^+^ microglia around the injured site at the indicated days after rmTBI. n = 6 for each experiment. ^##^p < 0.01 at 7 and 42 dpi compared with sham. Scale bar 50 µm (magnification ×200)

Intense signals for Iba-1^+^ were observed around the site of injury at 7 dpi (Fig. 3c). At 7 dpi, the percentage of Iba-1^+^ microglia was approximately 4 times more than that of 1 dpi (Fig. 3b). Then it declined at 14 dpi (Fig. 3d) and finally increased at 28 dpi (Fig. 3e) and 42 dpi (Fig. 3f). Quantitative data for the percentage of Iba-1^+^ microglia are shown in Fig. 3g.

**Fig. 4:**
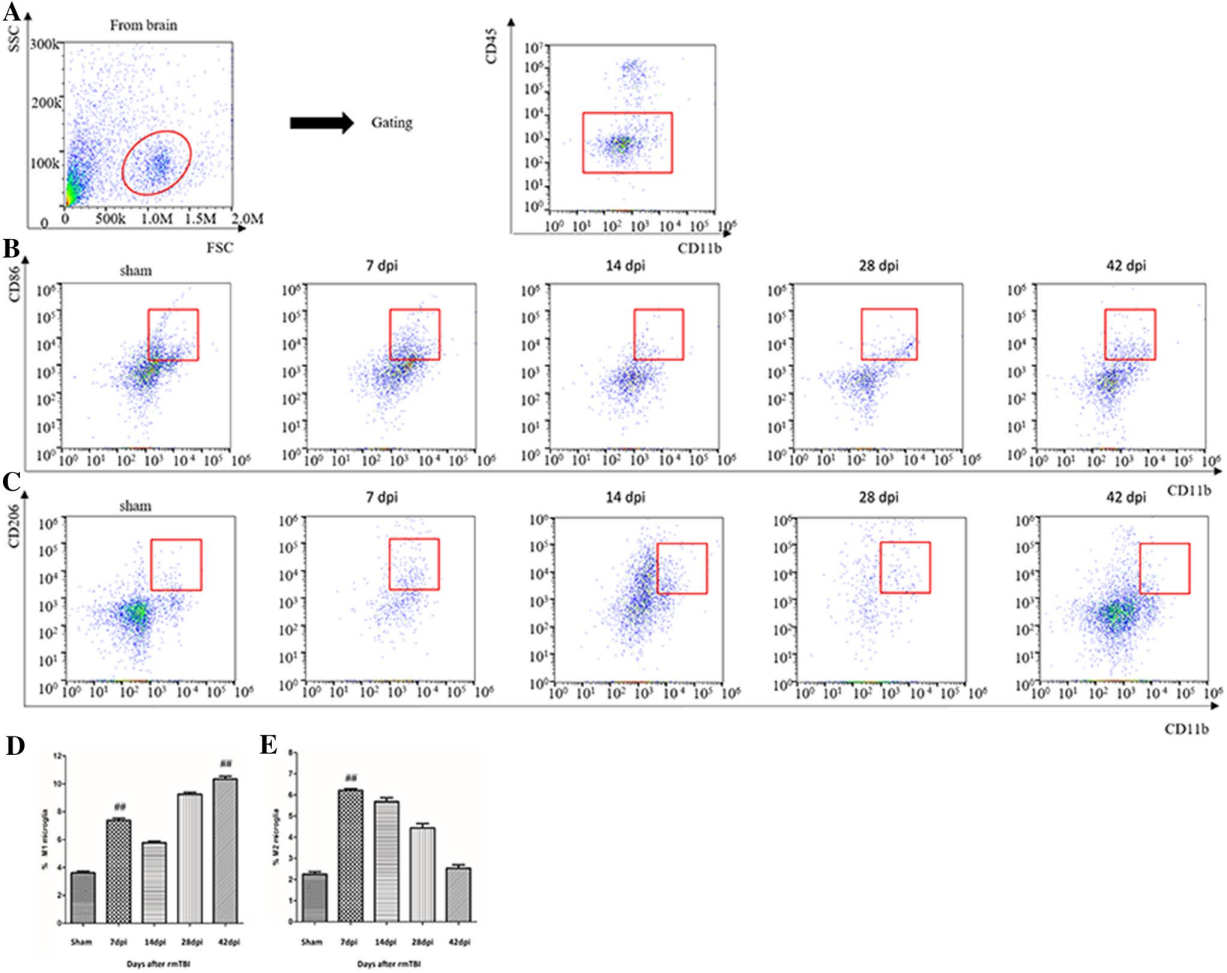
Analysis of subsets of microglia in the injured brain after rmTBI. **a** Representative flow cytometry data for the purity of microglia isolated. Of the isolated cells, 90% were microglia .**b** Representative flow cytometry data for CD86^+^/CD11b^+^ M1-like microglia in the injured brain at the indicated days after rmTBI. **c** Representative flow cytometry data for CD206^+^/CD11b^+^ M2-like microglia in the injured brain at the indicated days after rmTBI. **d** Graph illustrating quantitative data showing the percentages of CD86^+^/CD11b^+^ M1-like microglia. **e** Graph illustrating quantitative data showing the percentages of CD206^+^/CD11b^+^ M2-like microglia. n = 6 for each experiment. CD86^+^/CD11b^+^ M1-like microglia: ^##^p < 0.01 at 1 and 42 dpi compared with sham. CD86^+^/CD11b^+^ M2-like microglia: ^##^p < 0.01 at 7 dpi compared with sham

Two distinct populations of macrophages and microglia were observed: CD45^high^/CD11b^+^ cells and CD45^low^/CD11b^+^ cells. CD45^high^/CD11b^+^ cells were considered macrophages and CD45^low^/CD11b^+^ cells were considered microglia [3, 4]. Microglia have been classified into two subsets: pro-inflammatory M1 and anti-inflammatory M2 [5, 6]. CD86 is considered an M1 marker, and CD206 is considered an M2 marker [7]. Flow cytometry was performed to further examine the dynamic changes of distinct subsets of microglia in the brain. Of the isolated cells, microglia account for 90% (Fig. 4a). CD86^+^/CD11b^+^ M1-like microglia significantly increased at 42 dpi (Fig. 4b). CD206^+^/CD11b^+^ M2-like microglia peaked at 7 dpi and roughly returned to the baseline level at 42 dpi (Fig. 4c). Quantitative data for the percentage of M1 and M2-like microglia are shown in Fig. 4d, e.

### Dynamic Changes of T Lymphocyte Subsets in the Peripheral Blood After rmTBI in Rats

**Fig. 5:**
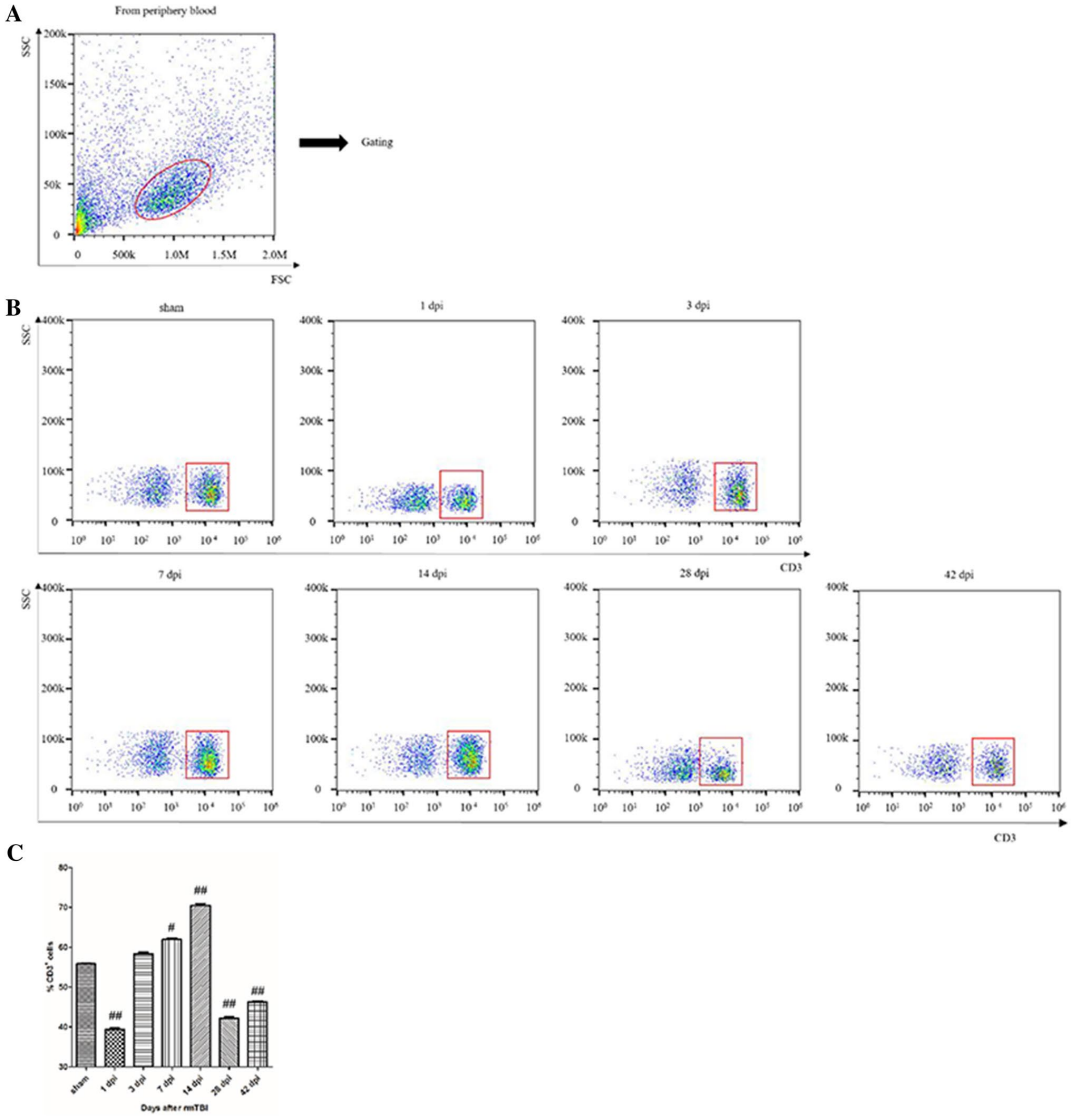
Analysis of T lymphocyte in the peripheral blood after rmTBI. **a** *Dot plots* of isolated immune cells in the peripheral blood, gated for live cell analysis. **b** Representative flow cytometry data for T cells (CD3^+^ cells) in the peripheral blood at the indicated days after rmTBI. **c** Graph illustrating quantitative data for accumulated T cells in the peripheral blood after rmTBI. n = 6 for each experiment. T cells: ^##^p < 0.01 at 1, 14, 28, and 42 dpi compared with sham, ^#^p < 0.05 at 7 dpi compared with sham

The gating strategy of live cell analysis was shown (Fig. 5a). The number of T cells decreased at 1 dpi, and rose slightly by 14 dpi, which again decreased at 28 and 42 dpi (Fig. 5b). Quantitative data for the percentage of T cells are shown in Fig. 5c.

**Fig. 6:**
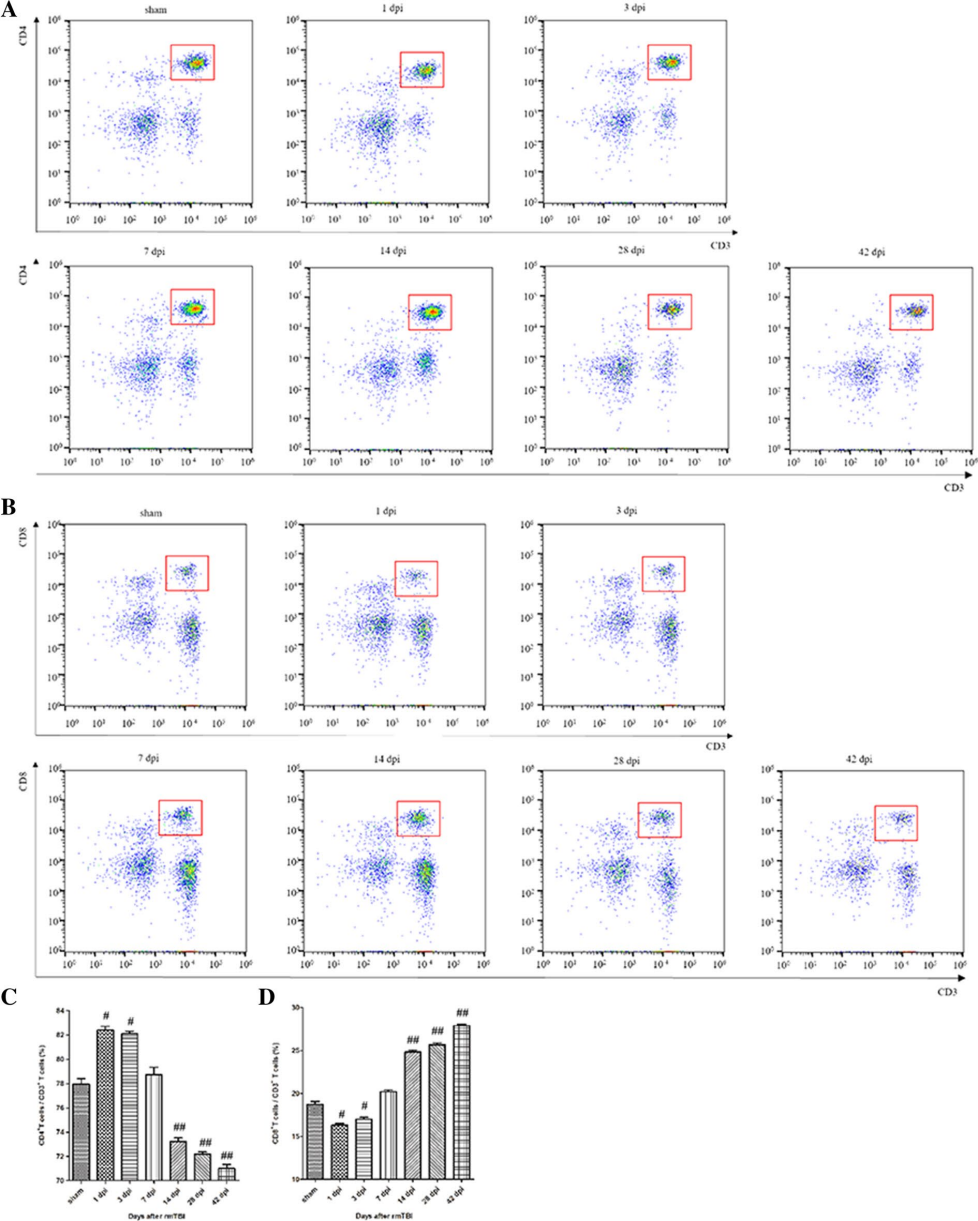
Analysis of T cell subsets in the peripheral blood after rmTBI. **a** Representative flow cytometry data for CD4^+^ T cells (CD3^+^ CD4^+^ cells) in the peripheral blood at the indicated days after rmTBI. **b** Representative flow cytometry data for CD8^+^ T cells (CD3^+^ CD8^+^ cells) in the peripheral blood at the indicated days after rmTBI. **c** Graph illustrating quantitative data for the percentages of CD4^+^T cells in the peripheral blood at the indicated days after rmTBI. **d** Graph illustrating quantitative data for the percentages of CD8^+^ T cells in the peripheral blood at the indicated days after rmTBI. n = 6 for each experiment. CD4^+^ T cells: ^##^p < 0.01 at 14, 28, and 42 dpi compared with sham; ^#^p < 0.05 at 1 and 3 dpi compared with sham. CD8^+^ T cells: ^##^p < 0.01 at 14, 28, and 42 dpi compared with sham; ^#^p < 0.05 at 1 and 3 dpi compared with sham

The percentage of CD4^+^ T cells continuously declined from 7 to 42 dpi (Fig. 6a), while the percentage of CD8^+^ T cells increased from 7 to 42 dpi (Fig. 6b). Quantitative data for the percentage of CD4^+^ and CD8^+^ T cells are shown in Fig. 6c, d.

## Discussion

T lymphocytes, a group of complicated multifunctional cell colonies, include many subsets of CD3^+^, CD4^+^, CD8^+^ and others. Representing T lymphocytes, CD3^+^ can reflect the condition of cellular immunity and immune response to different exogenous antigens. Regarded as T helper cells or T inducible cells, CD4^+^ can help B cells to promote the function of various antibodies. Defined as T suppression cells or T cytotoxic cells, CD8^+^ can help B cells to restrain various antibodies and exert cytotoxic effect on MHC-I antigen at the membrane of target cells. Treated as a crucial marker in immune regulation, the percentages of CD4^+^ T cells and CD8^+^ T cells are often recognized as a manifestation of immune balance [8]. In the injured brain, CD3^+^ T cells showed a bimodal increase during 42 dpi (Fig. 1). CD3^+^CD4^+^ T cells firstly increased and then decreased, while CD3^+^CD8^+^ T cells had reversed tendency (Fig. 2). Thus, there was alterations in T cell homeostasis in CNS after rmTBI in rats.

Microglia are resident immune cells in the brain, which regulate inflammatory response after a CNS injury. Changes in the ratio of pro-inflammatory M1 cells versus anti-inflammatory M2 cells reveal the direction of immune response [9, 10]. We observed that a bimodal increase at 7 and 42 dpi (Fig. 3), coinciding with the flow cytometry that CD206^+^/CD11b^+^ M2-like microglia peaked at 7 dpi, whereas CD86^+^/CD11b^+^ M1-like microglia increased at 42 dpi (Fig. 4). Given that M1-like microglia displays a primed role in the ageing CNS [11], we make reference, where appropriate, to modification of the M1/M2 balance in the immune system to provide plausible treatments following rmTBI.

Especially, we observed that the peak of T cells in the peripheral blood were at 14 dpi (Fig. 5), which showed a 7 dpi delay compared to that of microglia in CNS, consisting with Xuemei Jin’s view that T cells in the cervical lymph nodes respond to activated microglia in the injured brain transiently [12]. Hence, we observed that peripheral immunosuppression in the chronic phase after rmTBI (Fig. 6). Also, impaired CD4^+^/CD8^+^ T cell function was found in several studies on chronic injuries [13], and the immunosuppression could be lasted about 3 months [2, 14].

Generally, TBI immediately activates the SNS, leading to splenic contraction, spleen shrinkage [15], and dysfunction of immunity [16], all of which eventually make profound immune suppression. In later stages, because of exhausted spleen-derived immune cells and dramatically reduced spleen size, post-injury peripheral immunosuppression will develop [17]. Specifically, proposed mechanisms underlying peripheral immunosuppression impact on susceptibility of infections, which includes a set of molecular determinants [18] (high-mobility group box 1 [HMGB1] [19–21], ATP [22–24], S100 [25, 26]), activation of the HPA axis and SNS [27, 28], and disruption of the blood–brain barrier (BBB) [29].

In summary, what is characteristic of our finding is that dynamic changes of T cell subsets and microglia was implicated in the acute and chronic phase post-rmTBI. Further studies need to be explored, including the mechanisms underlying changes of the M1/M2 balance, the specific roles of CD3^+^/CD4^+^/CD8^+^ T cells in CNS or the peripheral blood, and the possible relationship between immunity and the post-injury prognosis.
